# Predicting the Lung Adenocarcinoma and Its Biomarkers by Integrating Gene Expression and DNA Methylation Data

**DOI:** 10.3389/fgene.2022.926927

**Published:** 2022-06-30

**Authors:** Wang-Ren Qiu, Bei-Bei Qi, Wei-Zhong Lin, Shou-Hua Zhang, Wang-Ke Yu, Shun-Fa Huang

**Affiliations:** ^1^ Computer Department, Jing-De-Zhen Ceramic Institute, Jingdezhen, China; ^2^ Department of General Surgery, Jiangxi Provincial Children’s Hospital, Nanchang, China; ^3^ School of Information Engineering, Jingdezhen University, Jingdezhen, China

**Keywords:** multi-omics data, feature selection, survival analysis, deep neural network, lung adenocarcinoma biomarkers

## Abstract

The early symptoms of lung adenocarcinoma patients are inapparent, and the clinical diagnosis of lung adenocarcinoma is primarily through X-ray examination and pathological section examination, whereas the discovery of biomarkers points out another direction for the diagnosis of lung adenocarcinoma with the development of bioinformatics technology. However, it is not accurate and trustworthy to diagnose lung adenocarcinoma due to omics data with high-dimension and low-sample size (HDLSS) features or biomarkers produced by utilizing only single omics data. To address the above problems, the feature selection methods of biological analysis are used to reduce the dimension of gene expression data (GSE19188) and DNA methylation data (GSE139032, GSE49996). In addition, the Cartesian product method is used to expand the sample set and integrate gene expression data and DNA methylation data. The classification is built by using a deep neural network and is evaluated on K-fold cross validation. Moreover, gene ontology analysis and literature retrieving are used to analyze the biological relevance of selected genes, TCGA database is used for survival analysis of these potential genes through Kaplan-Meier estimates to discover the detailed molecular mechanism of lung adenocarcinoma. Survival analysis shows that COL5A2 and SERPINB5 are significant for identifying lung adenocarcinoma and are considered biomarkers of lung adenocarcinoma.

## Introduction

Lung adenocarcinoma (LUAD) is one of the malignant tumors threatening human health and life, accounting for 40–50% of the total number of lung cancers (Ma et al., 2020). The occurrence of LUAD is inextricably linked with dietary habits, physical conditions, and environment. Studies have shown that the 5-years survival rate of patients with LUAD does not exceed 17% (Barrett et al., 2015; Chieh et al., 2016; Yang et al., 2016). At present, the most commonly used treatment methods for LUAD patients are radiotherapy and chemotherapy (Sun et al., 2011). The early symptoms of patients with LUAD are inconspicuous so they cannot be treated in time and may bring great pain to patients.

In recent years, the rapid development of modern bioinformatics technology can easily collect high-throughput omics data of various cancers, providing a new direction for cancer diagnosis. It is of great significance to understand the changes in cancer at the molecular level and find out biomarkers through omics data (Zhou et al., 2010; Chen et al., 2014). With the development of machine learning, artificial intelligence technology and assisted diagnosis can be applied to the research field of oncotherapy.

At the moment, many researchers have studied the pathogenesis of various cancers (Zhang et al., 2021) based on omics data. Using single omics data to find cancer biomarkers is not authoritative enough while multi-omics data can more comprehensively analyze the characteristics of the entire genomics (Park et al., 2020). However, the combination of different omics data requires mutual relationships to establish reliable and effective contact. Therefore, little research related to LUAD has been proposed by using multi-omics data. Furthermore, in machine learning, sparse samples with high dimensions usually cause larger errors. It is very important to select a subset of genes that distinguish phenotypes from high-throughput omics data. Studies have shown that effective feature selection can identify a subset of genes with high interpretation ability for the diagnosis of cancer (Rathore et al., 2014).

In this paper, we develop a model based on a deep neural network (DNN) by which one can predict LUAD using gene expression and DNA methylation data. At present, researchers have used multi-omics data sets to predict Alzheimer’s disease and confirmed the feasibility of this method (Park et al., 2020). However, the most challenging task is how to deal with high-dimensional and low-sample-size (HDLSS) data when the LUAD prediction model is constructed based on two different omics data sets. Because the biological characteristics of different omics data are different, common feature selection algorithms can not be used to reduce features. Therefore, we propose a biometric feature selection method to reduce the features and retain their biological significance. At the same time, we also compare other feature selection methods and machine learning algorithms. The experimental results show that the model proposed in this paper could obtain the best prediction performance. In addition, we also explore the relationship between potential genes and LUAD through gene ontology analysis, literature review, survival analysis, and find that COL5A2 、 SERPINB5 are the biomarkers of LUAD.

## Related Work

With the development of sequencing technology, the public genome database is becoming more and more complete. Through the analysis of data, genes play an important auxiliary role in the mechanism and prediction of cancer. The dimension of cancer gene data is much higher than the number of samples. The main work to deal with this problem is to reduce the dimension.

Feature transformation is a method to extract the features in the original space by some mapping transformation, such as PCA, PLS, etc. Using the new combined data to learn the classification model has achieved good classification results, but the biological significance is not clear. Feature selection is to evaluate the original genes to a certain extent and take out the features with good judgment ability to form a feature subset. The selected features have good explanatory power. Therefore, feature selection is a very effective method in high-dimensional data such as gene expression.

Liyingxin (Li et al., 2005) put forward the evaluation standard of “classified information index”. By removing irrelevant and redundant features to further narrow the selection range, five features are selected by SVM-RFE method, and high accurate recognition is achieved on the experimental dataset. Shipp (Shipp et al., 2002) used the signal-to-noise ratio method to select 30 features for classification of DLBCL data set (58 diffuse large B-cell lymphomas, 19 follicular lymphomas, 7,129 genes), and the correct recognition rate reached 91%. However, most of the above methods are based on some classification and evaluation criteria to judge the characteristics and select the genes with high scores. Because genes have similar expressions, they have similar prediction abilities in classification evaluation. If two of them are selected at the same time, the classification accuracy will not be improved, and it will also bring some redundancy problems. Park et al. (2020) proposed a biomarker prediction model to predict Alzheimer’s disease, which integrates multi-omics data. Experimental results have showed that their method has higher accuracy than using single data.

## Materials and Methods

**FIGURE 1:**
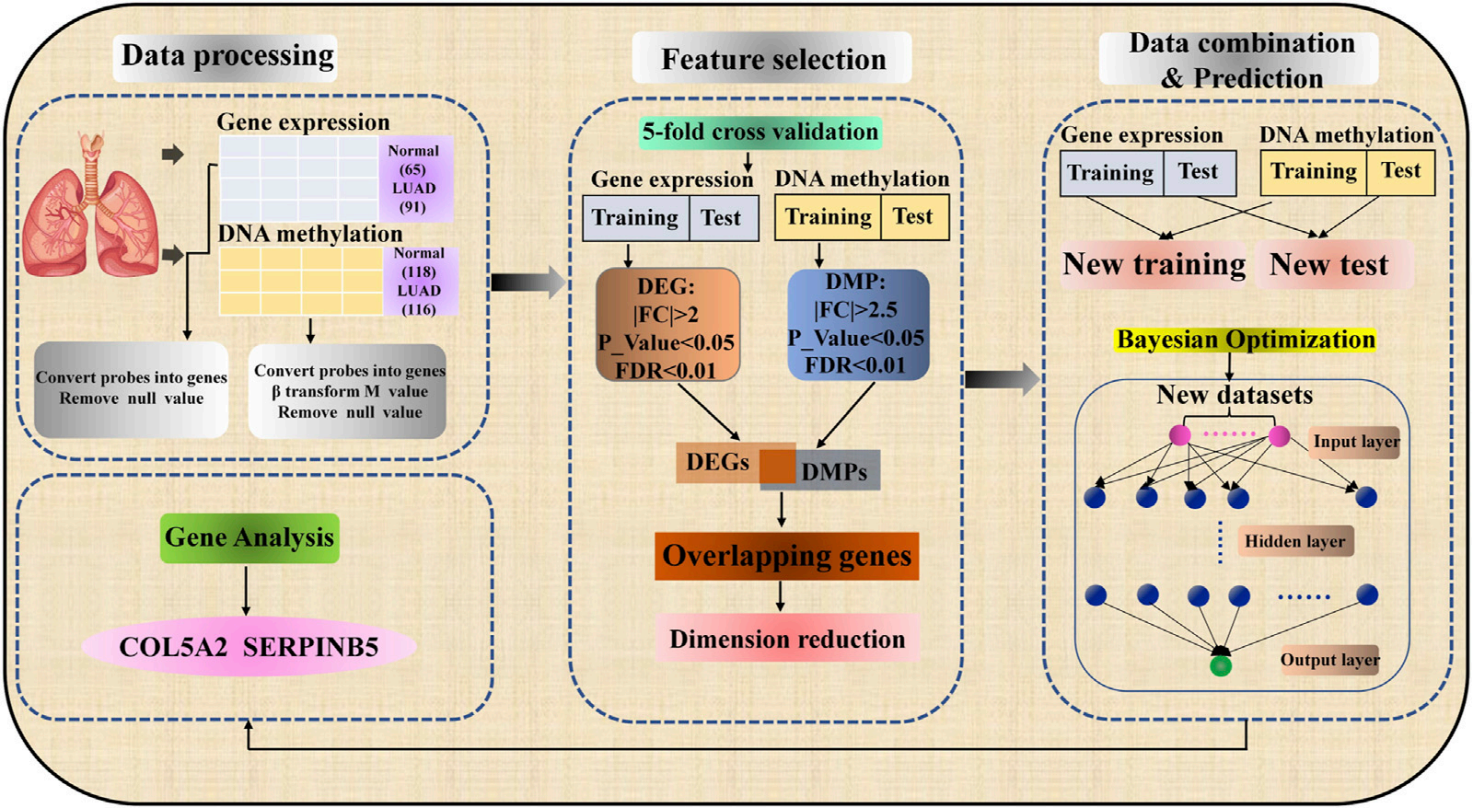
Workflow of lung adenocarcinoma prediction and biomarker recognition.

In this section, our work is to introduce the specific process of predicting LUAD and identifying biomarkers. As shown in Figure 1, the process mainly includes three parts: data preprocessing, feature selection, data combination, and prediction.

### Data Collection and Preprocessing

In this study, we download the microarray dataset of LUAD from the Comprehensive Gene Expression Omnibus of the National Center for Biotechnology Information (Edgar et al., 2002). Comparing microarray dataset with Next Generation Sequencing (NGS) dataset, we find that the sample size of LUAD dataset with NGS sequencing method in GEO database is very small, and the experiment will be very difficult due to high data dimensions and few samples (Mosele et al., 2020). Therefore, we analyze LUAD using the microarray dataset used by most researchers (Yan et al., 2012; Zhou et al., 2016). We collect two types of omics data, i.e., gene expression data and DNA methylation data. The Gene expression data can be obtained according to the following steps: 1) Enter the GEO database homepage https://www.ncbi.nlm.nih.gov/geo/, enter the keyword “lung adenocarcinoma” and click search. 2) Select “Expression profiling by array” in the “Study type” option, “*Homo sapiens*” in the “Top Organisms”. DNA methylation data is collected by the following standards: 1) Complete step one of gene expression data. 2) Select “Methylation profiling by array” in the “Study type” option, and “*Homo sapiens*” in the “Top Organisms”. 3) To expand the sample size, two DNA methylation data sets with sufficient sample sizes and the same platform are selected. According to the above criteria, gene expression data GSE19188 (Hou et al., 2010), DNA methylation data GSE139032 (Enfield et al., 2019), and GSE49996 (LuLu et al., 2015; Lenka et al., 2017) are obtained. In GSE4999, there are 41 normal samples and 39 tumor samples after the outliers were removed. Table 1 shows the detailed information of the benchmark dataset.

**TABLE 1:** Benchmark dataset.

Dataset	Gene expression	DNA methylation	
GEO ID	GSE19188	GSE139032	GSE49996
Normal samples	65	77	41
LUAD samples	91	77	39
Features	23,489	24,025	24,025

The data processing can be divided into three steps in this work. Firstly, the probes in the CEL file of gene expression data and DNA methylation data are transformed into genes according to the platform annotation file. When multiple probes correspond to the same gene, the average value is taken and the gene with a null value is deleted. Secondly, The R package “Affy” has been used to correct and standardize the data (Gautier et al., 2004). Different from the gene expression data, DNA methylation data need to convert Beta value to  M value after standardization, because M value is more suitable for statistical testing to determine the methylation ratio of each CpG sites (Du et al., 2010). The specific conversion is shown in Formula 1, where Beta is determined by calculating the intensity ratio between methylated and unmethylated alleles. It is a continuous variable between 0 and 1. When Beta≤ 0.2 is complete unmethylation, Beta≥ 0.6 is complete methylation, and 0.2≤Beta≤0.6 is partial methylation. Finally, the pre-processed data is divided into the training set and test set by the five-fold cross validation.

$$M=\log_{2}\left(\frac{Beta}{1-Beta}\right)$$ (1)

### Feature Selection Method

The gene expression data and DNA methylation data are the basis for constructing the prediction model of LUAD. These data sets have HDLSS characteristics. If the datasets with HDLSS characteristics are directly used to build the prediction model, there will be severe overfitting and high variance of gradient, which is the main challenge of machine learning. In order to solve this problem, we use the biometric selection method to reduce features and reduce the risk of overfitting. Identifying differentially expressed gene (DEG) is a typical method to process gene expression data, and the selected genes have clear biological interpretation (Porcu et al., 2020). Selecting only disease-related characteristic genes from all features can not only avoid the disaster of dimensionality but also effectively improve the classification effect. More importantly, differentially expressed genes can be used to study the mechanism of disease or as clinical biomarkers for early diagnosis. In DNA methylation data, DNA methylation can control the expression of genes near CpG sites, we mainly reduce the characteristics and retain the biological significance of the data set by identifying differential methylation position (DMP). FC (fold change) and *t*-test are used to calculate the differences in transcription levels between healthy and diseased individuals, so as to identify DEG or DMP which may be disease-related factors.

The principle of the FC algorithm is to calculate the multiple of the average expression level of genes in two types of samples. If the value reaches the preset threshold, it will be determined that the genes are differentially genes. The principle of *t*-test is to calculate a t-statistic for each gene to measure the difference in gene expression between the two types of samples, and then calculate the significance *p*-value according to the t-distribution to measure the significance of the difference. In addition, to prevent some genes from being misjudged as differential genes, we also calculate the false discovery rate (FDR) through the significance analysis of the microarrays algorithm to control the error rate of multiple tests and reduce the false-positive rate of results.

So far, many researchers have applied the method of identifying DEG and DMP to process gene expression data and DNA methylation data and identify cancer biomarkers more accurately (Li et al., 2019; Motalebzadeh and Eskandari, 2021). Faced with the complex gene relationship, Yang et al. argued that MI can effectively filter out pathogenic genes and provide a new way for drug repositioning (Yang and Hao, 2019). Dolezal et al. proposed a t-SNE model for dimensionality reduction, which can reliably distinguish all normal tissues and tumor tissues based on the characteristic RPT expression pattern (Dolezal et al., 2018). MI and t-SNE methods can reduce feature discovery of pathogenic genes, but cannot reflect biological processes, so they are not suitable for dimensionality reduction of multi-omics datasets.

The identification of DEG and DMP is achieved through the “limma” package of the R software (Ritchie et al., 2015; Maksimovic et al., 2016). For gene expression data to identify DEGs, the threshold value shall satisfy |logFC|>2, *p*-value<0.05, and FDR<0.01. In DNA methylation data, the same way is used to identify DMPs, and the threshold should meet | logFC | > 2.5, *p*-value < 0.05 and FDR <0.01. Genes are differentially expressed, hypermethylation and hypomethylation in different samples occurred. This gene may have a potential relationship with LUAD. As a result, it is reasonable to believe that the overlap between DEG and DMP has a potential relationship with LUAD, so we take the intersection of DEG and DMP (Peng et al., 2017).

### Data Combination and Operation Engine

In this paper, we take the integrated data as the input layer of the prediction model, so data combination is a key link. As shown in Figure 1, after feature selection, all possible gene expression data, and DNA methylation data of LUAD samples and normal samples are combined into a new data set by using the Cartesian product. Specifically, the new dataset was generated by combining data of gene expression and DNA methylation for normal and lung adenocarcinoma samples, respectively. New lung adenocarcinoma samples were obtained by combining lung adenocarcinoma samples with gene expression data and lung adenocarcinoma samples with DNA methylation data. For example, there are 65 normal samples and 91 LUAD samples for gene expression data, 118 normal samples and 116 LUAD samples for DNA methylation data. In the same way, the new data set has 7670(=65×118) normal samples and 10556(=91×116) LUAD samples. After the Cartesian product, the new data set not only expands the number of samples but also reduces the features, which overcome the HDLSS nature of omics data.

Compared with typical machine learning algorithms such as random forest (RF), K-Nearest Neighbor (KNN), Naïve Bayesian (NB), DNN model together with conventional machine learning algorithms have also been used to predict various biomedical phenotypes (Singh et al., 2021). At present, more researchers further improve the DNN model through a feature selection algorithm to make it shows very excellent prediction performance (Chen et al., 2019). As shown in the third part, i.e. Data combination & Prediction, of Figure 1, the structure of DNN is composed of input layer, hidden layer and output layer, and the layers are fully connected. The classification quality of DNN model is affected by its parameters, so it is very important to select appropriate super parameters. At present, there are many ways to optimize the hyperparameters of the network. We chose the Bayesian optimization algorithm, which will build a probability model by calculating the past evaluation results of the objective function to find the value that minimizes the objective function (Wu et al., 2019). Compared with grid search or random search, Bayesian optimization has higher parameter adjustment efficiency. This research uses 5–10 hidden layers, 250–350 nodes per layer, a learning rate of 0.01–0.2, and a dropout rate of 0.5–0.9 to find the optimal hyperparameter combination. In the DNN model, the loss function is used to predict the deviation between the output values and the actual values. It measures the performance of the algorithm in a single training sample. Cross entropy is a common cost function, which measured the average of all sample errors on the entire training set. The DNN model is implemented using the API of Google TensorFlow. The accuracy, F1 Score, and the area under the receiver operating characteristics (AUROC) are used to evaluate the classification results of the DNN model. The definitions of these evaluation indicators are as follows:

$$Accuracy=\frac{TP+TN}{TP+FP+TN+FN}$$ (2)

$$F1\;Score=\frac{2TP}{2TP+FP+FN}$$ (3)

## Results

### Data Quality Check and Differential Gene Identification

**FIGURE 2:**
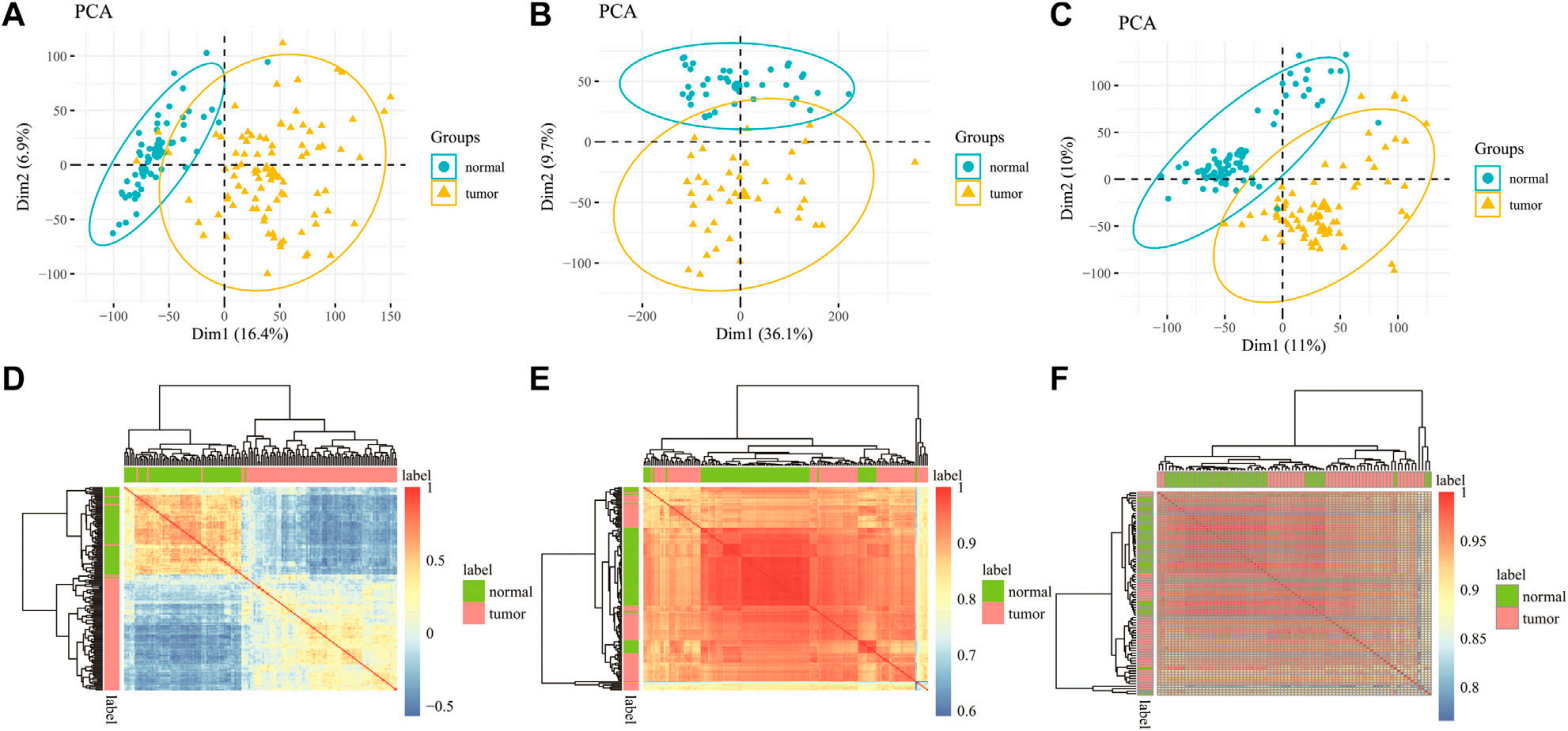
PCA for all samples in the **(A)** GSE19188 **(B)** GSE139032 and **(C)** GSE49996 dataset indicated two different groups. Pearson correlation matrix among all samples in **(D)** GSE19188 **(E)** GSE139032 and **(F)** GSE49996.

To verify the rationality of the data, we do principal component analysis (PCA) and Pearson correlation analysis, which respectively show the distribution of the data and the correlation between the samples to judge whether the data is feasible. In the PCA diagram, a point represents a sample. The farther the distance between two points, the greater the difference between the two samples. Figures 2A–C are the PCA diagrams of GSE19188, GSE139032, and GSE49996 datasets respectively. In the figure, normal samples and tumor samples gather in different regions and are far away. These results show that there are obvious differences between LUAD samples and normal samples, and the data distribution is good. Pearson correlation coefficient is used to express the correlation of samples. The value is between - 1–1. When the value is closer to 0, the correlation is lower, and the value is closer to - 1 or 1, the correlation is higher. As shown in Figures 2D–F are Pearson correlation analysis diagrams of samples in the data of gene expression data and DNA methylation data. The correlation coefficients between samples in the diagram are unequal, but this does not mean that there is a causal relationship between samples, and there are few samples with correlation coefficients of 1 or -1, which indicates that there is no repeatability between samples. From the results of PCA and Pearson correlation analysis, it can be seen that the selection of data is meaningful.

**FIGURE 3:**
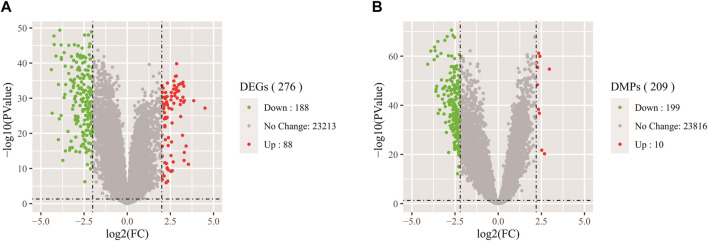
**(A)** Volcano map of DEGs in gene expression data. **(B)** Volcano map of DMPs in DNA methylation data.

A volcano figure can help us intuitively identify genes with large changes and statistical significance. As shown in Figures 3A,B, the volcanic plot is drawn according to gene expression data and DNA methylation data respectively. Each point in the map represents a detected gene, the red point represents the up-regulated gene, the green point represents the down-regulated gene, and the gray point represents the genes with no significant difference. In Figure 3A, the outside of the two black vertical lines are genes with |logFC| > 2, and the upper side of the black horizontal line are genes with a *p*-value less than 0.05. From the vertical axis, the farther away from the horizontal axis, the smaller the *p*-value, and the more significant the gene difference. As can be seen from the figure, the gene expression data includes 88 up-regulated genes and 118 down-regulated genes, which were the focus of our attention. In Figure 3B, the outside of the two black vertical lines is CpGs with |logFC| > 2.5. It can be seen from the figure that there are 209 CpGs differentially expressed in DNA methylation data, including 10 up-regulated CpGs and 199 down-regulated CpGs. These CpGs are reliable and more suitable for later identification of whether they are markers because DNA methylation can control the expression of genes near CpGs, thus affecting embryonic development and tumorigenesis.

### Experimental Design

In this paper, we propose the following three hypotheses: The accuracy of predicting LUAD using multi-omics datasets is higher than that using a single omics dataset; In the prediction of LUAD, the feature selection method using biometrics (DEG + DMP) is better than the ordinary dimensionality reduction algorithm; The performance of using DNN classifier to predict LUAD is better than that of the traditional classifier.

We divide the experiment into three sections to test the aforementioned hypothesis. I) Compare the accuracy of the prediction model by inputting different types of datasets. The DNN prediction model’s input dataset is separated into three types: gene expression, DNA methylation, and integrated dataset. II): Using MI, t-SNE, and biometric feature selection method to reduce the dimension of data. It is worth noting that the data should be reduced to the same dimension while utilizing different dimensionality reduction methods. III): When predicting LUAD, the performance of the traditional classifier (RF、KNN、NB) is compared with the DNN model. We used the dimension reduction algorithm provided by Scikit-learn package and the traditional machine learning algorithm.

The input layer of the DNN classifier in this article is the integrated gene expression data and DNA methylation data. Because it is dealing with binary classification problems, the output layer consisted of a node. It can be seen from the result of Bayesian parameter determination that the hidden layer is composed of eight layers, ReLU is introduced as the activation function, and each layer is composed of 300 nodes and a bias node. When training the model, adds one dropout to each layer, and the dropout rate is 0.85. DNN is compared with three classifiers of random forest (RF) (Cutler et al., 2001), K-Nearest Neighbor (KNN) (Hassanat et al., 2014), and naïve Bayesian (NB) (John, 1995). The specific experimental parameters are shown in Table 2.

**TABLE 2:** Parameter setting.

Methods	Parameter setting
DNN	learning rate = 0.02, dropout = 0.85
RF	criterion = 'entropy', n_estimators = 100, n_jobs = -1, max_depth = 6
KNN	n_neighbors = 10
NB	default parameters

### Comparison Between Single Omics Data Set and Multi-Omics Data Set

A total of nine combinations are offered to evaluate the effects of different feature extraction methods on prediction results: MI-gene expression, t-SNE-gene expression, DEG-gene expression; MI-DNA methylation, t-SNE-DNA methylation, DMP-DNA methylation; MI-integrate multi-omics data, t-SNE-integrate multi-omics data, DEG + DMP- integrate multi-omics data.

To avoid overfitting on the training set, when using single omics data set as the input dataset, we do five-fold cross validation of gene expression data and DNA methylation data. For each fold, the number of DEGs and DMPs identified from the training set is different. Therefore, when performing MI and t-SNE on each fold gene expression and DNA methylation dataset, we reduce the dimension to the same number of features as those obtained by DEG or DMP methods (as shown in Table 3). Because the training set of each folding input is different, the number of genes identified each time will be different. On average, the DEGs and DMPs decreased to 53 and 120.4 respectively.

**TABLE 3:** Dimensions of different feature extraction algorithms in each fold data.

5-Fold CV	Num of genes (MI t-SNE DEG)	Num of CpGs (MI t-SNE DMP)	Num of genes and CpGs (MI t-SNE DEG + DMP
K = =1	47	109	156
K = =2	52	120	172
K = =3	55	121	176
K = =4	57	131	188
K = =5	54	121	175
Avg	53	120.4	173.4

When a multi-omics dataset is used as an input dataset, the same as the process of using single omics data, MI and t-SNE algorithms are used to extract the same dimensionality as the feature algorithm proposed in this research, and the average value of the simplified dimensionality is 173.4. Traditional classifiers such as RF, NB, and KNN are selected for prediction. Table 4 shows the performance comparison of different prediction algorithms. It can be seen that the results of the feature selection method used in this paper are better than using MI or t-SNE in different data sets. For example, when the gene expression data after DEG feature selection is used as the input data set of the KNN prediction model, the accuracy of predicting LUAD is 0.9435, which is 0.411 higher than that of t-SNE and 0.0142 higher than that of MI. When the DNA methylation data after DMP feature selection is used as the input data set of the NB prediction model, the accuracy of predicting LUAD is 0.9435, which is much higher than that using MI and t-SNE feature selection methods. In particular, the results of using multi-omics data to predict LUAD are higher than using gene expression data or DNA methylation data.

**TABLE 4:** The Accuracy and AUROC results of different prediction algorithms.

		Gene expression			Methylation expression			Gene expression and DNA methylation		
		MI	t-SNE	DEG	MI	t-SNE	DMP	MI	t-SNE	DEG + DMP
RF	ACC	0.8387	0.5448	0.9435	0.5044	0.5766	0.9465	0.5000	0.5726	0.9659
	AUROC	0.8653	0.5489	0.9407	0.5053	0.5889	0.9377	0.5000	0.6209	0.9694
KNN	ACC	0.9293	0.5325	0.9435	0.5641	0.5171	0.9571	0.6132	0.4974	**0.9778**
	AUROC	0.9290	0.4516	0.9469	0.5667	0.5559	0.9459	0.6065	0.5171	**0.9780**
NB	ACC	0.6714	0.5065	0.9354	0.5044	0.6196	0.9659	0.5000	0.6099	0.9750
	AUROC	0.6264	0.4542	0.9317	0.5000	0.6192	0.9579	0.5000	0.5964	0.9652

### The Impact of Different Classifiers on Performance

To achieve the optimal performance of the DNN model, the Bayesian optimization algorithm is used to determine the optimal hyperparameters of the DNN model. Before model training, Bayesian optimization is performed on the integrated data set to obtain a parameter combination with the highest training accuracy. Finally, the average value of each parameter in the five-fold is applied to our model, and the optimal hyperparameters are shown in Table 2.

**FIGURE 4:**
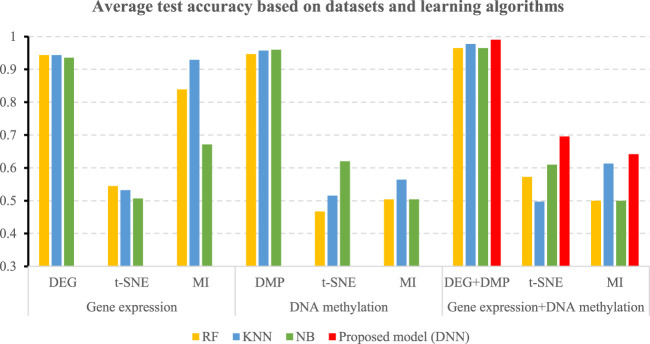
Average accuracy of all comparisons and the proposed model.

**FIGURE 5:**
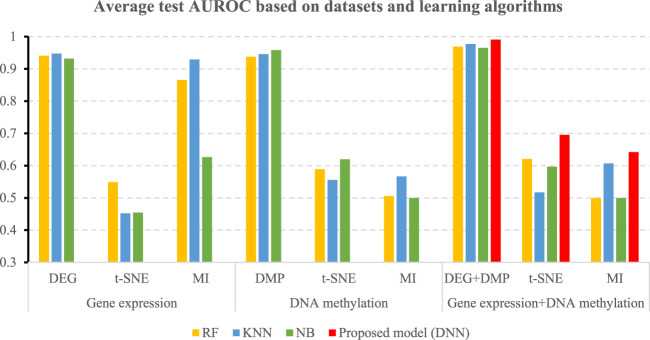
Average AUROC of all comparisons and the proposed mode.

**FIGURE 6:**
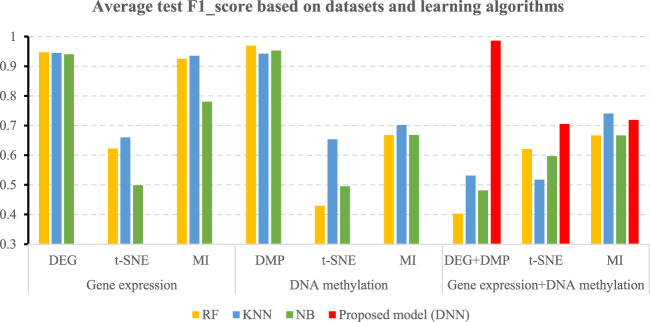
Average F1-score of all comparisons and the proposed mode.

Table 5 shows the accuracies and the values of the AUROC of the DNN model with different feature selection methods. Five-fold cross validation is also used to obtain the average values of accuracy and AUROC. It can be seen from the table that the average accuracy of the DNN model is 0.9903 when the feature selection method for identifying differential genes is used. To compare the performance of different classifiers more intuitively. Figures 4–6 have shown the average accuracy, average F1 Score, and AUROC of five-fold cross validation respectively. In the figure, X-axis presents the dimension reduction or feature selection approaches for single-omics and multi-omics datasets. Each color bar represents different classifiers, especially the red bars that indicate the performance of the proposed DNN model. Figure 4 demonstrates that the method based on deep learning showed the best accuracy in the integrated omics data set. Figure 5 shows the AUROC of the prediction model under different classifiers. After changing the data set and feature selection method at the same time, it shows that when the integrated omics data is used as the input data set of the DNN model, the AUROC is 0.9916, which is better than all comparison methods. Figure 6 demonstrates the F1_score of the prediction model under different feature selection methods and data sets. In all comparisons, the proposed DNN shows the highest F1_score, indicating that the model is the most robust. Currently, Pan et al. established a prediction model of LUAD with a support vector machine algorithm to study LUAD. The model takes GSE19188 data as the input data set, and the prediction accuracy was 0.97 (Pan et al., 2019), and Liu Kou et al. Determined the most valuable factors of LUAD metastasis through Kaplan-Meier survival curve and multivariate logistic regression analysis and constructed a metastasis prediction model. The average accuracy of the prediction model was 0.86. (Liu et al., 2019). In summary, these predictions are lower than the results of this study.

**TABLE 5:** 5-fold performance comparison of different feature selection algorithms in the deep learning-based prediction model.

	Mi			t-SNE			The proposed model		
	Cost	Accuracy	AUROC	Cost	Accuracy	AUROC	Cost	Accuracy	AUROC
1	1.9900	0.6156	0.4309	0.6508	0.7143	0.5000	0.0004	0.9999	0.9998
2	3.3951	0.6230	0.5000	0.6574	0.6230	0.5000	0.0171	0.9959	0.9967
3	6.7629	0.4298	0.5000	0.8742	0.4551	0.5077	0.1822	0.9849	0.9808
4	1.4862	0.5076	0.5028	0.9059	0.4702	0.4677	0.1213	0.9764	0.9772
5	3.2534	0.6534	0.5000	0.5965	0.6754	0.6193	0.0454	0.9945	0.9958
Average	3.3775	0.5659	0.4867	0.7370	0.5876	0.5189	**0.0732**	**0.9903**	**0.9916**

The average Cost, Accuracy and AUROC of DEG+DMP feature selection algorithm are used in the prediction model based on deep learning.

## Discussion

Table 6 shows the overlapping genes that meet DEG and DMP at the same time in each fold during five-fold cross-validati. We also select the genes that appear in each fold, to avoid the error caused by chance in crcross-validationFoninally, 12 key genes are identified and considered as potential biomarkers of LUAD. To prove whether these 12 genes are capable of recognizing LUAD, the following gene analysis has been made.

**TABLE 6:** Selected genes from gene expression and DNA methylation and comparison with LUAD database.

Each Ffold	K = 1	K = 2	K = 3	K = 4	K = 5
Selected genes	ABCA3 AIM2 CA3 CDKN2A COL1A1 COL5A2 CYYR1 FOXF1 GREM1 HLF MAGEA6 PCSK1 PROK2 SCNN1B SERPINB5 SLC7A11 SOSTDC1 SOX17 SOX7 STXBP6 TWIST1	ABCA3 AGTR1 AIM2 AZGP1 CA3 COL1A1 CLIC3 COL5A2 CYYR1 FOXF1 FOXF2 GDF10 GREM1 HLF MAGEA6 MAL MUC1 PROK2 S100A2 SERPINB5 SLIT3 SOSTDC1 SPARCL1 STXBP6 TRHDE TWIST1	ABCA3 C1orf116 COL1A1 COL5A2 COX7A1 CYYR1 FOXF1 GREM1 HIST1H2BH HK3 HLF MAGEA6 MAL MUC1 S100A2 SCNN1B SERPINB5 SLC7A11 SOSTDC1 SOX7 SPARCL1 STXBP6 TWIST1 ZBED2	ABCA3 AGTR1 C1orf116CDKN2A COL1A1 COL5A2COX7A1 CYYR1 EFEMP1 FOXF1 GDF10 GREM1 HLF IL6 MAL MMP13 PKP1 SERPINB5 SLC7A11 SOSTDC1m SOX7 SPARCL1 STXBP6 TWIST1	ABCA3 AZGP1 C1orf116 COL1A1 COL5A2 COX7A1 CYYR1 EFEMP1 FOXF1 GREM1 HLF MAGEA6 MMP13 PCSK1 PROK2 S100A2 SERPINB5 SLC7A11 SOX7 SPARCL1 STXBP6 TWIST1 ZBED2
Union of the selected genes across 5 fold			ABCA3 COL1A1 COL5A2 CYYR1 SLC7A11 GREM1HLF SERPINB5 SOX7 SPARCL1 STXBP6 TWIST1

The selected gene set is analyzed by using the DAVID database. As shown in Table 7, gene ontology reported the biological significance of genes. “GO:0050900 leukocyte migration” affects the proliferation and migration of LUAD A549 cells; “GO:0046982 protein heterodimerization activity” is related to LUAD (Zhou et al., 2017) and is also a similar biological activity in liver cancer (Wang et al., 2011) and nasopharyngeal carcinoma. The results showed that the activity of protein dimer interacted selectively and non-covalently with different proteins to form heterodimer (Lan et al., 2014); “GO:0001228 transcriptional activator activity, RNA polymerase II transcription regulatory region sequence-specific binding” is one of the important pathways involved in LINC00648, and is related to lung cancer (Zhao et al., 2020).

**TABLE 7:** GO analysis of selected genes.

Category	Term	*p*-value	Gene
GOTERM_BP_DIRECT	GO:0030198∼extracellular matrix organization	0.0001	COL1A1, FOXF2, FOXF1, COL5A2, SERPINB5, JAM2
GOTERM_BP_DIRECT	GO:0050900∼leukocyte migration	0.0042	COL1A1, SLC7A11, COL5A2, JAM2
GOTERM_BP_DIRECT	GO:0001558∼regulation of cell growth	0.0194	SOCS2, FAM107A, AGTR1
GOTERM_MF_DIRECT	GO:0001228∼transcriptional activator activity, RNA polymerase II transcription regulatory region sequence-specific binding	0.0287	SOX17, SERPINB5, FOXF1
GOTERM_MF_DIRECT	GO:0046982∼protein heterodimerization activity	0.0399	TWIST1, COL5A2, JAM2

After literature retrieving, the gene ABCA3, studies have shown that it is usually highly expressed in the damaged lung (Stahlman et al., 2007), and may provide important clues for the diagnosis of LUAD. THBS gene may play a double-edged sword role in the development, the anti-angiogenic and oncogenic function of LUAD. COL5A2 is one of the seven genes co-expressed by TSHB2. When COL5A2 is highly expressed, the survival rate of patients decreases (Weng et al., 2016). Compared with normal lung tissue cells, the SERPINB5 gene is specifically expressed at high levels in lung cancer cells and can be used as a diagnostic marker of lung cancer (Yoon et al., 2011). COL1A1 is considered a downstream product of cytoglobin, which is related to tumor biology and contributes to the adaptive response to oxidative stress and hypoxia/reoxygenation events, thereby promoting lung tumor invasiveness, metastasis, and resistance to treatment (Mendoza et al., 2015). Liu et al. proposed that COL1A1 is a potential biomarker for the prognosis of LUAD (Liu and Huang, 2020). Lung tumors rely on glucose, cystine, and glutamine. SLC7A11 is a cystine/glutamate transporter, which promotes tumor growth and development (Lin et al., 2020). Ji et al. also proposed that SLC7A11 overexpression is a candidate biomarker SLC7A11 for lung cancer (Ji et al., 2018). TWIST1 is involved in embryogenesis and promotes malignant transformation and LUAD progression through epithelial-mesenchymal transition (Karine et al., 2012).

**FIGURE 7:**
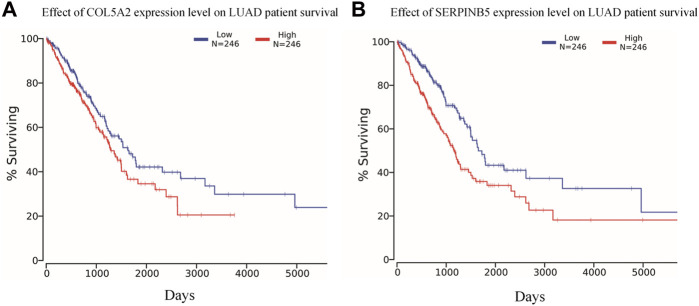
Overall survival analysis in LUAD based on the TCGA data as determined by Kaplan-Meier estimates. **(A)** COL5A2 and **(B)** SERPINB5 are significantly affect the prognosis of LUAD in overall survival (*p* < 0.05).

The Kaplan-Meier survival curve analysis of the 12 potential biomarkers identified in 492 LUAD patients using the TCGA database shows that COL5A2 and SERPINB5 are significantly correlated with the prognosis of LUAD (Figure 7).

## Conclusion

In this study, we use the DNN model to predict LUAD and identify biomarkers based on integrated multi-omics data. By comparing different feature selection methods and different prediction models, the results show that the method of this research is better. The advantage of the method is that it used integrated multi-omics data; there is no blind dimensionality reduction in feature selection, but biologically significant features are selected. The results of biological correlation analysis and literature verification also show that the selected genes can be used as biomarkers of LUAD. Although we have done careful bioinformatics analysis, there are still some limitations. In the future, we can continue to explore the application of this method to other similar omics data, and we will continue to improve our method.

## Data Availability

The datasets presented in this study can be found in online repositories. The names of the repository/repositories and accession number(s) can be found in the article/Supplementary Material.
